# Spaced radiology: encouraging durable memory using spaced testing in pediatric radiology

**DOI:** 10.1007/s00247-019-04415-3

**Published:** 2019-05-16

**Authors:** Cara E. Morin, Jason M. Hostetter, Jean Jeudy, Wendy G. Kim, Jennifer A. McCabe, Arnold C. Merrow, Alan M. Ropp, Narendra S. Shet, Amreet S. Sidhu, Jane S. Kim

**Affiliations:** 1 St. Jude Children’s Research Hospital, 262 Danny Thomas Place, Memphis, TN 38105 USA; 2 Department of Diagnostic Radiology & Nuclear Medicine, University of Maryland School of Medicine, Baltimore, MD USA; 3 Department of Diagnostic Radiology, Boston Children’s Hospital, Boston, MA USA; 4 Center for Psychology at Goucher College, Baltimore, MD USA; 5 Cincinnati Children’s Hospital Medical Center, University of Cincinnati College of Medicine, Cincinnati, OH USA; 6 Department of Diagnostic Radiology and Nuclear Medicine, University of Virginia School of Medicine, Charlottesville, VA USA; 7 Department of Diagnostic Imaging and Radiology, Children’s National Health System, Washington, DC USA; 8 Department of Internal Medicine, St. Joseph Mercy Oakland, Pontiac, MI USA

**Keywords:** Internet, Medical education, Memory, Picture archiving and communication system, Pediatric radiology, Self-testing, Spaced repetition

## Abstract

Applied memory research in the field of cognitive and educational psychology has generated a large body of data to support the use of spacing and testing to promote long-term or durable memory. Despite the consensus of this scientific community, most learners, including radiology residents, do not utilize these tools for learning new information. We present a discussion of these parallel and synergistic learning techniques and their incorporation into a software platform, called Spaced Radiology, which we created for teaching radiology residents. Specifically, this software uses these evidence-based strategies to teach pediatric radiology through a flashcard deck system.

## Introduction

Radiology trainees are presented with a large volume of factual knowledge and imaging patterns that must be readily recalled every time a new study is interpreted. This knowledge cannot be simply learned for board exams, but rather it needs to be stored for future recall during a lifetime of clinical service. Is there an ideal way to assimilate all of this information? Applied memory research in the field of cognitive and educational psychology has generated a large body of data to support three main learning strategies that consistently show benefits for long-term memory acquisition: elaboration, testing and spacing [1–4].

The initial step in learning requires encoding (i.e. forming memories of) information previously unfamiliar to the learner. In medical school and residency, this is usually performed by a combination of attending lectures, participating in small group sessions, and reading textbooks or journal articles. To facilitate the creation of long-term memories during this initial stage, learners use a variety of strategies they have developed over time. Most frequently, these include rereading notes or original texts and highlighting or underlining key passages. These strategies have been demonstrated to be relatively shallow and ineffective for longer-term learning [2, 5, 6].

Elaborative learning refers to deep processing of information at the encoding stage, where depth refers to greater semantic connection [7]. In practice, the various strategies under this category generally involve pausing from consuming information (whether by reading or listening) to ask why or how the material connects to one’s existing knowledge. For instance, while reading a review article on neonatal bowel gas patterns, the learner may consider examples they have encountered in practice, or question why the article states that the infant may have distal bowel gas in the case of malrotation with volvulus despite the presence of a proximal obstruction. A large body of data supports the superiority of various elaborative learning strategies across a variety of ages and ability levels as compared to more shallow types of processing [1–3]. However, elaborative strategies are less commonly used by individual learners during independent study (compared to relatively shallow strategies such as rereading or highlighting) for several reasons, including a lack of awareness of evidence-based study strategies and presumed time constraints [1, 5, 8–10].

Research suggests that both testing and spacing help to further strengthen memory after the initial encoding stage by allowing forgetting and encouraging effortful recall from long-term memory. This article will review these parallel and synergistic techniques by first describing the goals of these strategies. Then we will present a progress update on a new platform employing these evidence-based strategies for image-based pattern learning in pediatric radiology. This initiative was funded by the Society for Pediatric Radiology (SPR) Research and Education Foundation (REF) Education Project Grant.

### Testing/retrieval-based learning

Self-testing, or retrieval-based learning, produces the most robust learning and long-term retention of material in experimental models [2, 4–6, 11, 12]. This is the concept that engaging in effortful retrieval (i.e. testing) of information from long-term memory results in superior memory outcomes compared to rereading or restudying. Testing can refer to flashcards, practice problems/questions, or low-stakes (and even no-stakes, i.e. unscored) practice tests.

Self-testing as a superior learning strategy is supported by both laboratory studies and real-life trials in undergraduate students, medical students and residents. For example, a study involving medical students showed that the short-term recall of medical facts was significantly improved in a group using retesting flashcards (i.e. cues were provided with the answer on the opposite side of the card) versus restudying flashcards (cues and answers provided on the same side of the card) [11].

Not surprisingly, additional data support the concept that an increased amount of practice affects the efficacy of self-testing on memory and long-term recall. Thus, learners who repeat flashcards until they answer correctly multiple times, not just once (also called high criterion learning), demonstrate improved short- and long-term recall [6].

Multiple studies have demonstrated that both undergraduates and medical students lack awareness of the testing effect when surveyed about their study practices [5, 6, 11–13]. That is to say, students who endorse self-testing as a study strategy do so to assess their level of knowledge to guide further studying and not because self-testing itself can promote durable memory for later recall. Of course, identifying areas to focus further study is itself a valuable result of self-testing. However, this lack of awareness appears to be evolving, with one newer study demonstrating that a substantial number of medical students report self-initiated retrieval practice using a flashcard-based method [14]. This mirrors findings from undergraduates: In an older study, a minority of students endorsed testing over rereading material [8], yet a newer study showed an increased percentage of students choosing testing as a more effective strategy [15].

### Spaced learning/distributed practice

**Fig. 1:**
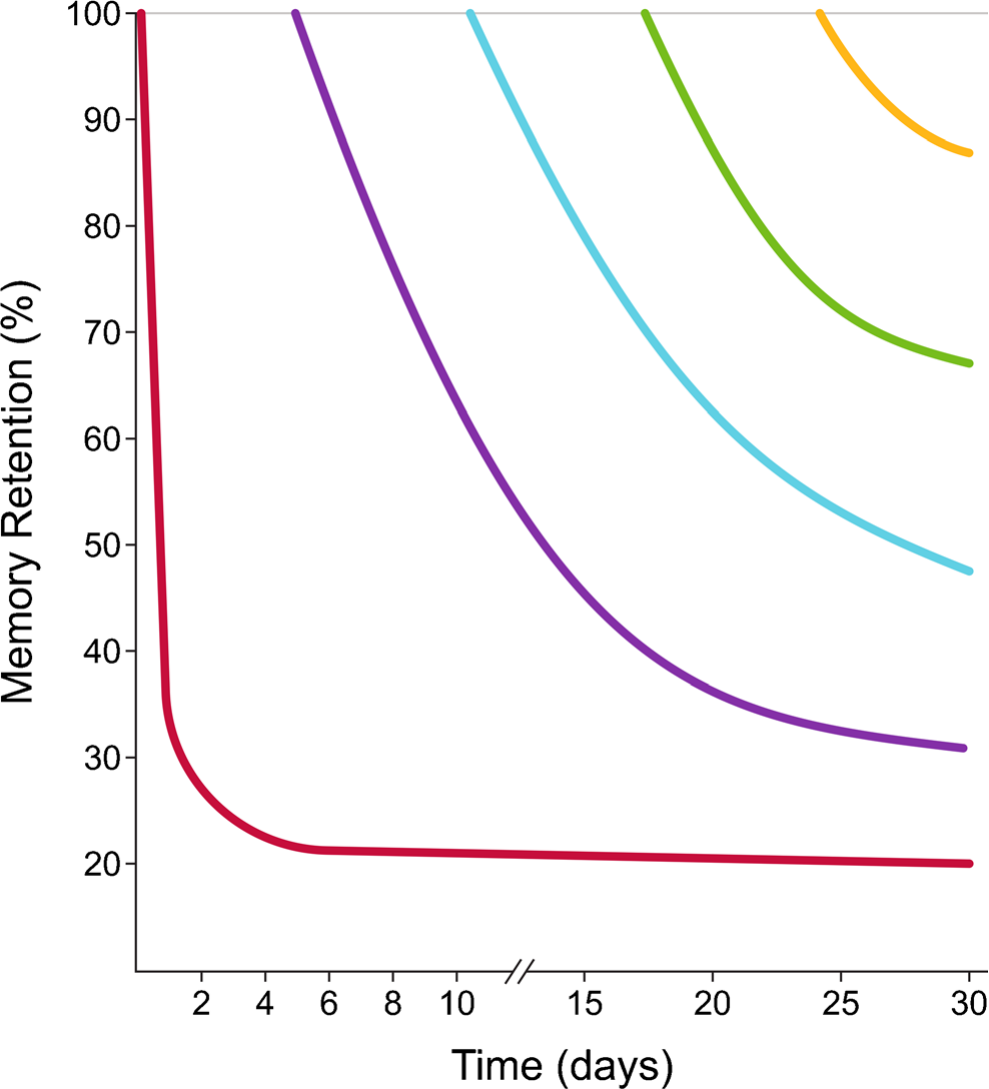
Adaptation of the “forgetting curve” initially described by Ebbinghaus in 1885 [16]. The red curve is a typical representation of a forgetting curve, showing that memory retention falls exponentially after initial encoding of new information. Active, effortful strategies of learning are required to interrupt the decline. The purple, blue, green and yellow curves show the hypothetical impact of repeated study/test sessions at later dates, with each forgetting curve less steep than the one prior

One of the oldest and most established methods for enhancing memory is known as the spacing effect (or distributed practice), initially described by Ebbinghaus in 1885 [16–18]. From this early research, we know that forgetting happens over time and that most of it happens within a day or so of initial learning, as illustrated by the “forgetting curve” (Fig. 1). Active, effortful strategies, such as spacing and testing, interrupt the decline in the forgetting curve, allowing for more successful long-term retention of information. Hundreds of experiments have confirmed the superiority of distributed practice versus massed/blocked practice (“cramming”) (reviewed in [19]). Available data support the notion that, while massed practice can speed the acquisition of knowledge, the acquired material is not necessarily retained long term. Rather, it is far superior to space study sessions over time to enhance memory. Despite the huge volume of research supporting this, many undergraduate and medical learners do not practice this method [8, 9] nor is it encouraged or implemented by institutions. Technology-driven educational platforms have the opportunity to interrupt the forgetting curve by prompting practice at various intervals: frequently after encoding and then at longer intervals over time [20].

Many of the studies examining spaced learning have compared study schedules of shorter versus longer interstudy intervals. Research suggests that long-term retention improves with longer intervals between study sessions [2]. This has been demonstrated specifically using self-testing as the study strategy, which is also more effective when using longer vs. shorter lag times between practice trials, both within and between sessions [6].

### Evidence-based learning methods in medical education

Over the past decade, there has been increasing attention paid to incorporating the above methods in medical education, more often for medical students than for postgraduate trainees (i.e. residents or fellows) [4, 14, 21–26]. A study by Deng et al. [14], queried all medical students at their institution following the United States Medical Licensing Examination (USMLE) Step 1 exam for self-reported use of various study methods with or without spaced repetition and found that the majority of students used a flashcard-based spaced repetition program. They also found that higher scores correlated with those students who reviewed more cards in a specific program (Anki; https://apps.ankiweb.net/) [14].

There are more than 15 freely available digital programs that allow learners to make and share flashcards [27]. A recently published article provides a guide for medical students to establish collaborative digital flashcard projects using free software that incorporates the principles of active recall and spaced repetition [28]. Commercial products harnessing flashcard and spaced education techniques have proliferated recently, including Firecracker (Wolters Kluwer, Alphen aan den Rijn, The Netherlands) [29] and Qstream (Burlington, MA) [30]. Further, open-source resources, such as the Brosencephalon (https://www.brosencephalon.com/) flashcard collections (utilizing Anki), have emerged as popular options in the toolkit of many trainees worldwide [31]. These platforms harness the power of distributed practice by prompting learners to complete practice sessions at various intervals. Notably, all of the available platforms are limited to static information and do not incorporate interactivity such as image scrolling or zooming.

Few published studies have focused on postgraduate learners [21, 32–34]. Larsen et al. [21] performed a randomized controlled trial to evaluate the effect of repeated testing on the retention of information taught in a pediatric neurological emergency conference for pediatric and emergency medicine residents. Their results demonstrate that retesting produced better long-term retention of the information learned from a didactic conference relative to repeated, spaced study, including testing up to 6 months after the date of the conference [21]. Going beyond increased clinical knowledge, Dolan et al. [33] were able to demonstrate improvements in patient care for residents participating in a novel adaptive online curriculum in the area of bone health.

### Current use of e-learning tools in radiology

Radiology education has drastically changed in the last two decades since the introduction of digital picture archiving and communication systems (PACS). The digital format of image acquisition uniquely positions radiology to seamlessly integrate images into resident education. Development of computer-based learning tools for radiology has been recognized as a useful adjunct to resident education since at least 1995 [35]. Traditional PACS-mediated teaching files are generally restricted to within an institution, while other tools such as the Radiological Society of North America (RSNA)-supported Medical Imaging Resource Community have not gained widespread use.

Radiology residents continue to migrate away from textbooks to online learning tools, particularly in the setting of the new computerized multiple-choice American Board of Radiology (ABR) Core exam. The most utilized study resources for the Core exam, per an annual chief resident survey (2015), were RSNA online physics modules and RadPrimer, with both at greater than 90% usage among all surveyed 3rd-year residents [36]. Moreover, the number of commercial online question bank websites has rapidly increased since the advent of the Core exam, with at least six such websites advertising to radiology residents [37].

In a 2014 survey of radiology residents and staff, residents utilized Google and resident-generated study materials more often than any other resource, including textbooks and radiology journals [38]. To answer specific imaging questions, radiology residents most often reported using Google or STATdx and rarely other resources such as PubMed, textbooks or individual journals. Overall, the results of the survey indicated that both radiology residents and staff are moving away from hard-copy textbooks and journals in favor of online and mobile resources at a rapid rate [38].

Outside of diagnostic radiology residency, a web-based platform for teaching oral radiology to dental and dental hygiene students using spaced repetition platform was shown to be easy to implement and well-liked by students [39].

Despite the proliferation of online question banks specifically targeting radiology residents studying for the ABR Core exam, there remains a lack of image-rich tools that utilize the established strategies of spacing and testing to teach radiology in general, including pediatric radiology. The majority of digital programs that allow learners to make and share flashcards did not until recently allow for the addition of images to the cards and even now allow only static images (e.g., JPEGs [Joint Photographic Experts Group]). None of the currently available radiology quiz platforms incorporates any spacing algorithm to prompt practice sessions.

### Pilot data

A prototype version of the flashcard deck concept was developed and tested among residents in our residency program (Morin CE, Hostetter J, Ropp A et al., unpublished data). The deck included 84 cards with images of adult chest radiographs in a PACS-like viewer. Nine diagnoses (atelectasis, emphysema, fibrosis, heart failure, lung mass, mediastinal/hilar adenopathy, pleural effusions, pneumonia and pneumothorax) as well as normal radiographs were included. All diagnoses were confirmed by contemporaneous CT imaging. Eighteen residents reviewed the flashcard deck once and all answers were recorded. Subsequently, residents attended a 2-week block of daily 1-h didactic thoracic radiology lectures as part of the standard residency curriculum, including a lecture specifically covering the relevant diagnoses included in the quiz. On the last day of lectures, residents again reviewed the flashcard deck and answers were recorded. A survey was also administered at both time points.

The average score across residents rose by 10% between the flashcard review sessions, a small increase, which is not surprising. A single review of flashcards and didactic lectures would not be expected to result in a significant increase in pattern recognition and memory of diagnoses. However, the accompanying survey demonstrated that 100% of the responding residents believed the flashcard method would improve their ability to recognize and diagnose the included chest radiograph abnormalities, and all responding residents recommended the deck be reviewed before starting independent call.

### Filling in the gap

In the spring of 2016, we were awarded the SPR REF Education Project Grant based on a proposal to create a platform for the development and administration of interactive image-rich quizzes to teach pediatric radiology. Our goal was to incorporate evidence-based strategies for long-term memory retention, focusing on self-testing with optimization of criteria and spacing. We also took into consideration lessons from the work of previous authors who developed electronic quizzes or radiology simulator programs. These authors have identified ideal characteristics for a quiz format in radiology [40–42]. This left us with the following project goals:

1. Closely approximate the appearance and functionality (scrolling, window/leveling, measuring, magnifying, etc.) of a PACS. The ability to manipulate images is a major advantage over other image-rich quiz programs because it simulates the real-life experience of clinical interpretation [42].
2. Accept fully anonymized HIPAA (Health Insurance Portability and Accountability Act)-compliant studies in DICOM (Digital Imaging and Communications in Medicine) format (allowing for the previously mentioned PACS functionality). Case entry should be simple and preferably automated.
3. Be accessible by mobile technology (both iOS and Android compatible) reflecting data that radiology residents primarily use online resources for studying.
4. Provide both immediate (i.e. showing the correct diagnosis at the time of studying) and long-term (i.e. allowing the learner to discover areas of weakness over time) feedback to the learner.
5. Include normal studies in addition to abnormal studies, mirroring a normal clinical workflow and introducing the learner to the spectrum of normal exams. Additionally, including normal studies changes the dynamic for the learner to first decide if the study is normal or abnormal and then identify the abnormality.
6. Utilize a spaced repetition algorithm.

**Fig. 2:**
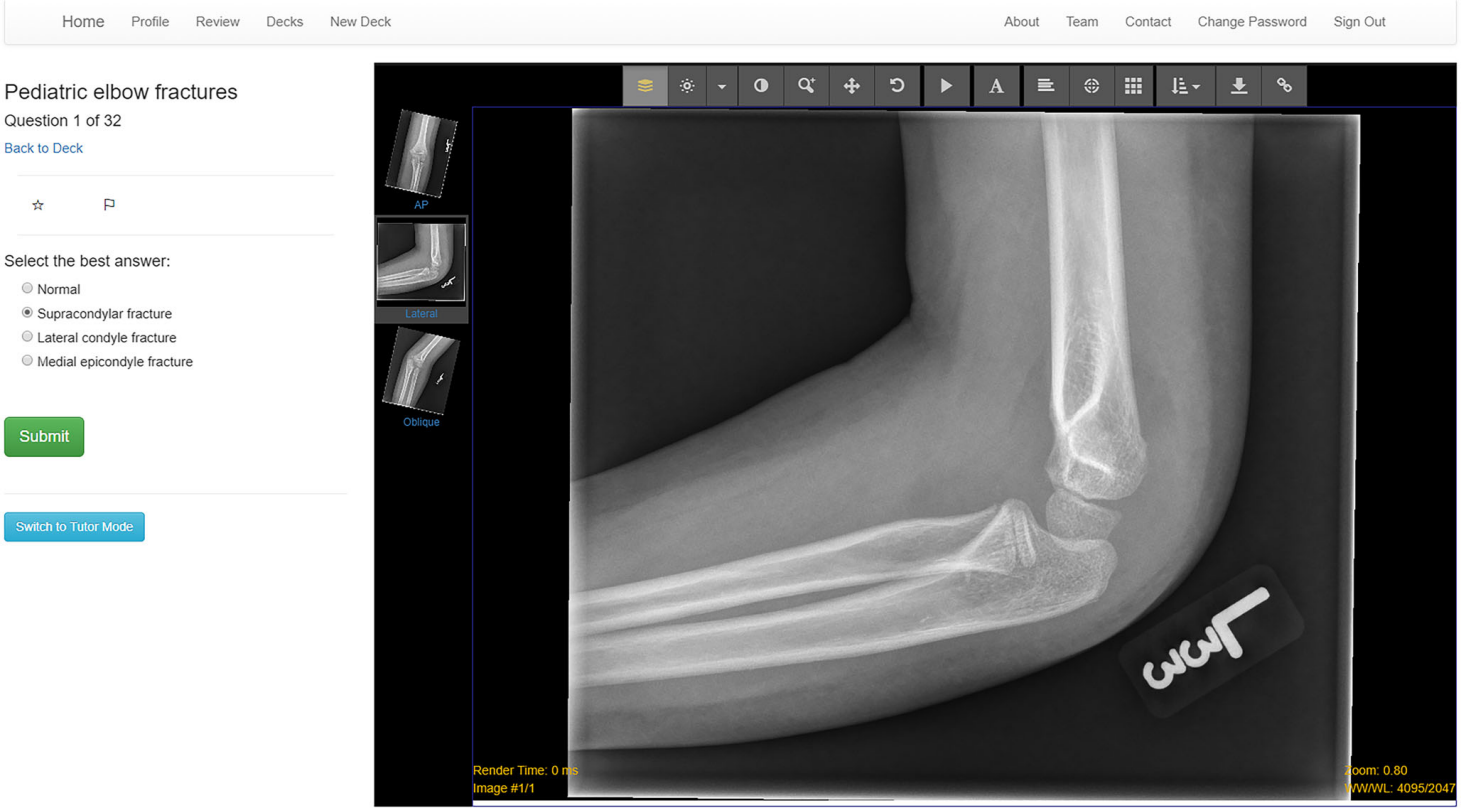
This screenshot demonstrates the user interface of one flashcard in a deck. This card demonstrates the limited number of diagnoses included in each deck (in this case four, including normal). Additionally, the integration with Pacsbin is demonstrated, in this case showing three images from one radiograph series

This led to the creation of a website called Spaced Radiology (http://www.spacedradiology.com/), which hosts a collection of flashcard decks (Fig. 2). Each deck contains a set of images/cards collated by specific themes, such as The Limping Child. Learners complete individual decks and then the cards are sorted by a spaced retrieval algorithm. Several aspects of Spaced Radiology were designed to explicitly align with evidence-based strategies from applied memory research. These are described below.

### Encouraging effortful retrieval/testing

To increase the efficiency of practice sessions, multiple examples of each diagnosis are included in each deck (which is also known as high criterion learning). For example, multiple different cases of supracondylar fractures are mixed into a deck covering the topic of elbow fractures. This serves to improve memory retention and pattern recognition via repetitive exposure to force repeated retrieval until a high level of accuracy is obtained, and to expose the learner to slightly different cases of the same diagnosis, so the learner develops a strategy to categorize patterns of diagnoses. Research suggests interleaved practice of exemplars from multiple categories is superior for category learning, as compared to massed/blocked practice of one type of exemplar at a time [43]. In other words, including examples of cases from pediatric chest radiology and pediatric musculoskeletal radiology in the same study session should improve durable memory compared to studying each subject separately. Normal cases are also included in many decks, which allows the learner to encounter the wide range of normal variants, and forces the learner to decide whether an abnormality exists.

### Incorporating spaced repetition

**Fig. 3:**
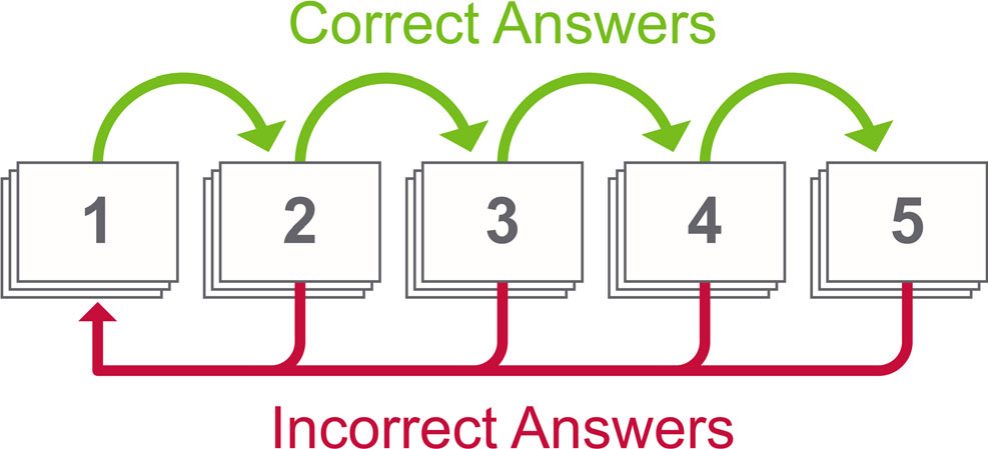
The Leitner system is a spaced repetition algorithm, which sorts flashcards into five piles according to how well the learner knows each diagnosis. Each correct answer advances the flashcard to the next, less frequent pile. Incorrect answers are sent back to the first pile. This system skews exposure toward unknown diagnoses

Evidence shows that increased lag time between learning targets is associated with improved longer-term memory retention [19]. The very nature of high criterion learning means that multiple examples of diagnoses are presented, which inherently increases spacing between cards. Additionally, we implemented the Leitner system, one of the most popular algorithms for spacing of flashcards, which skews exposure toward unknown diagnoses [44]. The structure of the Leitner system forces the user to review incorrect material at higher frequencies than known diagnoses by stratifying tested concepts into piles (Fig. 3). Currently, our quiz modules contain between three and five diagnoses with four to six examples of each diagnosis. Though research suggests studying larger decks produces more spacing and therefore more durable learning as compared to smaller decks [45], preliminary testing in the current system has shown that learners prefer shorter decks with fewer than 30 cards.

### How it all works in practice

DICOM upload and integration with the platform is accomplished by leveraging Pacsbin.com (Orion Medical Technologies, LLC, Baltimore, MD), a cloud-based anonymized PACS platform for education and research. The Pacsbin platform enables DICOM studies to be embedded into other websites using an API (application programming interface) [46]. The embedded studies provide full window/level capabilities, annotations, scrolling and other expected functions provided by standard PACS systems.

DICOM images are uploaded to the cloud Pacsbin platform using an integrated export pipeline triggered through the institutional PACS system. The export pipeline performs study anonymization and upload to the cloud. At the time of export, the case can be annotated with notes, tagged and sorted into collections.

When the user is ready to make a deck, a study is imported to a Spaced Radiology card via a web link. Full image data sets can be included within a card using this process, allowing users to view entire studies with PACS-like manipulation. Alternatively, cards can be created using JPEG images; however, those cards do not have PACS functionality. Images can be annotated with regions of interest to denote abnormalities, which can then be tied to quiz questions.

When a learner is reviewing a deck, the answer choices reflect the limited number of diagnoses in the deck, and the order of answer choices does not change, allowing the learner to focus on the image. The learner can choose to either see the answer after each card or to wait to see all the answers at the end of the deck. Once a learner has finished an individual deck, the cards are sorted into a “space repetition review” pile, which includes all the cards taken to date.

### Submission of quizzes/peer review

**Fig. 4:**
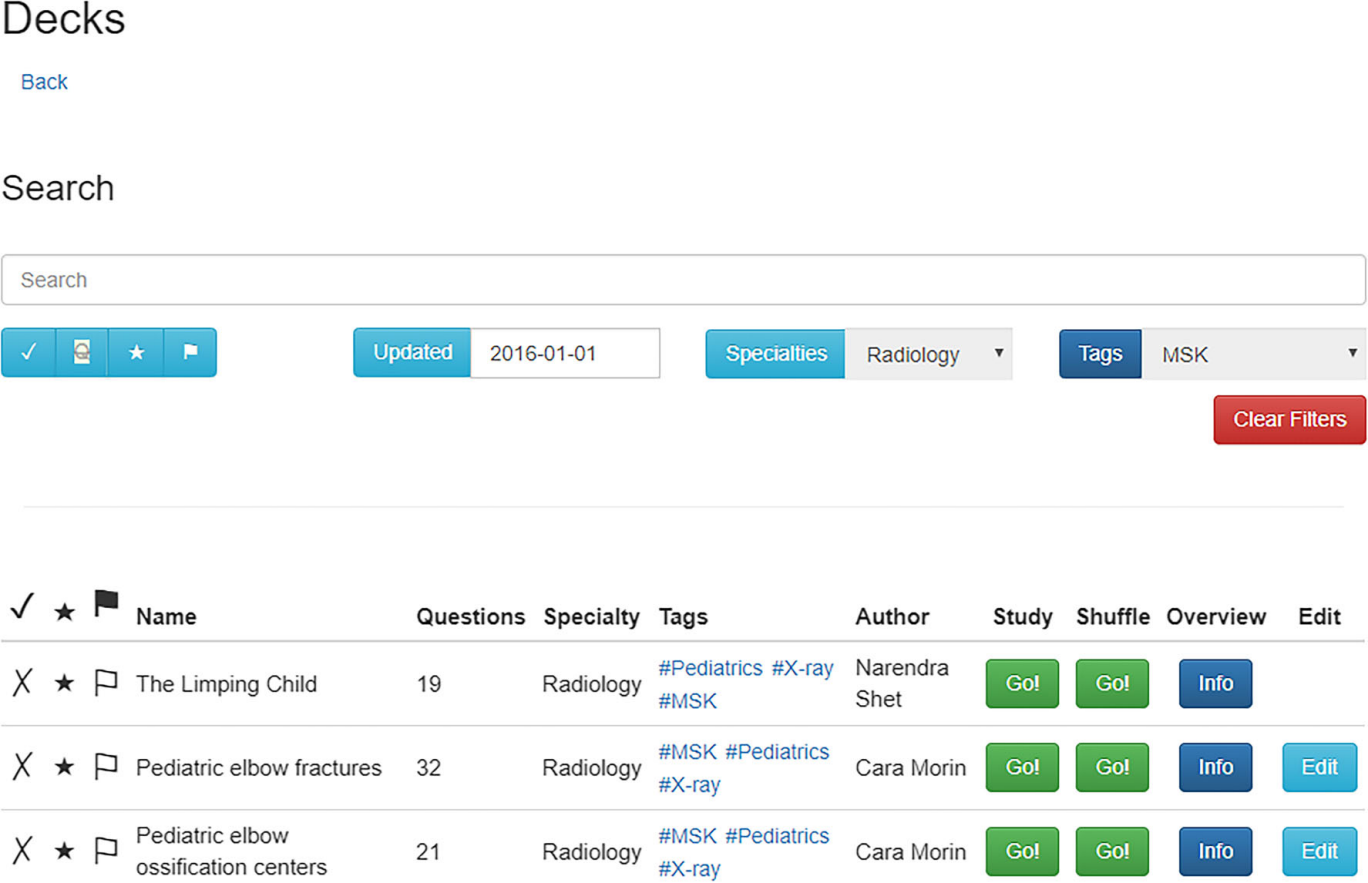
This screenshot demonstrates the use of filtering on the deck search page. Available flashcard decks can be filtered by those with administrative approval, individual user flags, specialty, tags (such as musculoskeletal [MSK] in this example) or author

In the platform’s current form, any user can make a quiz that can be shared with other users. To create the best education content, we have incorporated a peer review system for quality control, which allows for reviewed content to be featured with a seal of approval. The peer-reviewed deck must be approved by an administrator-level account. After approval, the deck is featured on the deck search page, easily found by other learners (Fig. 4). If a deck is not peer reviewed, it is only searchable as non-reviewed content.

For our pediatric decks, we have a group of academic pediatric radiologists who serve as reviewers. This ensures excellent quality images and accuracy. We plan to identify additional experienced radiologists to serve as reviewers for other fields in radiology.

Ten decks have been created as part of the initial platform development, with subject matter including Neonatal Chest, The Limping Child, Salter-Harris Fractures, Pediatric Elbow Ossification Centers, Pediatric Elbow Fractures, Hip Avulsion Fractures, NICU (neonatal intensive care unit) Baby Bellies, Viral Bronchiolitis? and VCUG (voiding cystourethrography) Reflux Grading. The current decks were assembled by individual radiologists and then reviewed by at least two additional radiologists for consensus approval of included cases to focus on ensuring excellent quality images, a range of appearances of the selected diagnoses (e.g., inclusion of a spectrum of supracondylar fractures from subtle to fairly obvious), inclusion of a range of ages when applicable, and agreement on the underlying diagnosis. When possible, clinical information, follow-up cross-sectional imaging or pathology reports were utilized for confirmation of diagnosis. In the absence of definitive diagnosis, consensus opinion of three attending radiologists was deemed acceptable.

**Fig. 5:**
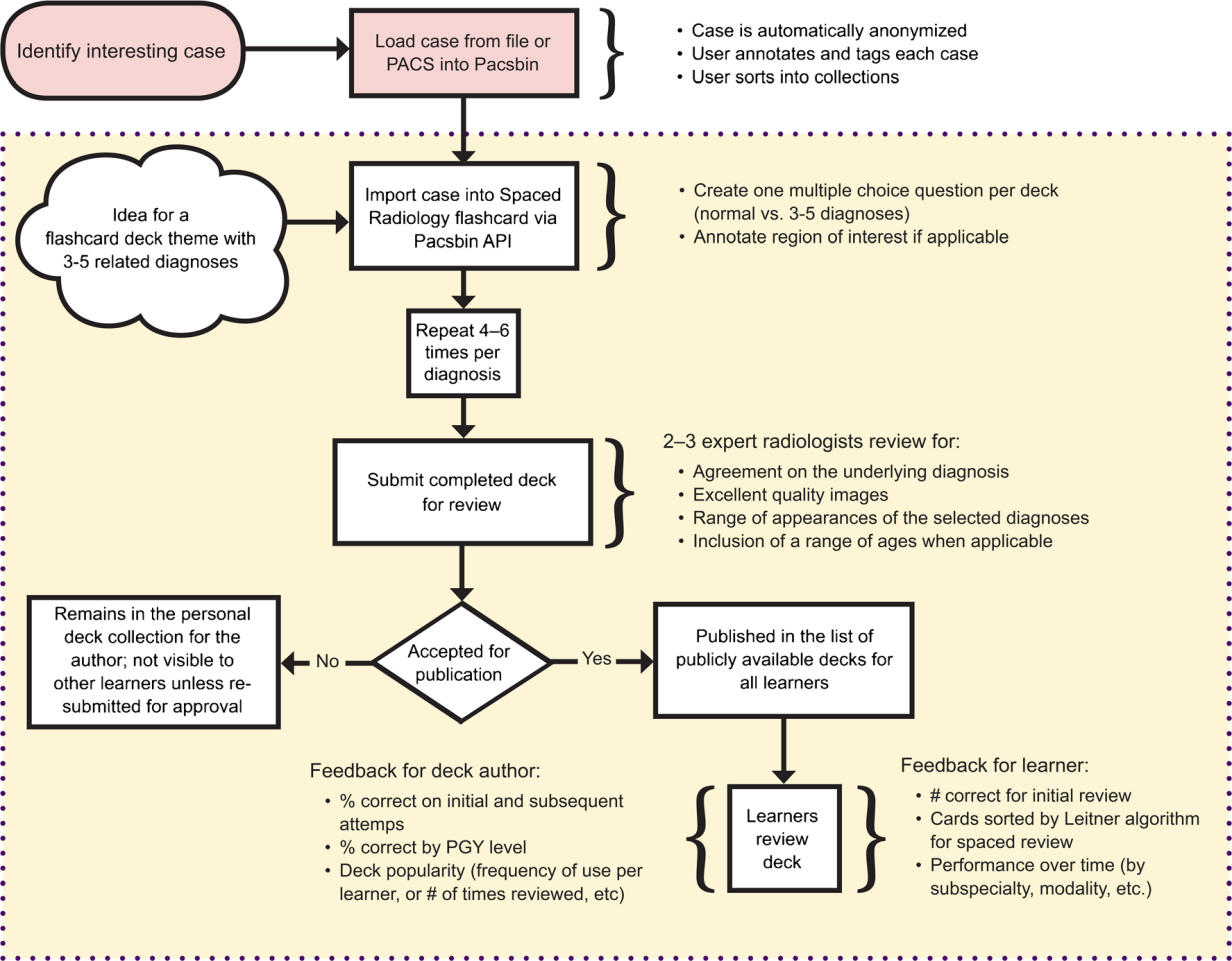
This diagram of the workflow from identifying an image for saving in Pacsbin to the ultimate product of a flashcard deck. All of the steps within the dashed box represent the innovations of the Spaced Radiology platform

The time it takes to create a new deck varied widely depending on the topic and individual radiologist habits of case curation in Pacsbin. Once all cases were collected and reviewed for quality, the creation of a deck in Pacsbin can be finished in less than an hour. The process of creating a deck is diagrammed in Fig. 5.

### Outcomes data

Using this system, a large amount of data will be available for both evaluation of learners as well as evaluation of individual cards and decks. Learners will be able to monitor their own progress, including the number of cards used and performance over time. We propose that as learners use the platform and their performance improves, their appreciation of the testing effect will increase.

Data will be collected for deck use by topic, performance over time and performance by postgraduate year, among other outcomes. Deck popularity may be a good indication of the usefulness of included topics.

Advances in performance tracking of individual and groups of learners will allow us to determine the efficacy of our testing strategies. We plan to optimize the ideal number of diagnoses per deck (for the best intra-deck lag time) and the ideal lag time between review sessions by administering several variations of the available decks to radiology residents with official testing sessions at various delays to evaluate long-term retention.

## Discussion

We describe the development of a platform that harnesses both spacing and testing to implement an image-based pediatric radiology curriculum. Spacing and testing are both examples of a category called desirable difficulties [47, 48]. These are encoding strategies that are slow and effortful at the time of initial learning, and can even lead to errors but are superior for long-term memory retention. Learners often do not realize that desirably difficult strategies contribute to durable learning, as they can seem nonobvious or even counterintuitive [8]. Using what we know from applied memory research to teach pediatric radiology, our program utilizes the learning methods that will most enhance long-term memory [1].

In addition to implementing evidence-based strategies for durable memory, a secondary benefit of creating sets of images to review is the standardization of radiology education. On any given rotation, a radiology resident may encounter a limited number of diagnoses, which will be different than the following resident’s diagnoses the next week or month. Rare diagnoses may not be seen at all on a service or at an institution. The ability to see different cases representing common and rare diagnoses in radiology is instrumental in allowing the learner to recognize the diagnosis in the future [41].

In addition to decks based on simple pattern recognition that we have created thus far, the platform could be adapted for more complex diagnoses, such as congenital heart disease. A deck with multiple examples of cardiac computed tomography (CT) or magnetic resonance imaging (MRI) scans demonstrating post-Fontan anatomy, for example, would be useful for radiology residents who train in hospitals without a pediatric cardiothoracic surgery program.

Furthermore, trainees and practicing physicians outside of radiology often require basic knowledge of many imaging patterns. For example, pediatric emergency department physicians often need a basic proficiency in interpreting chest and extremity radiographs for common diagnoses, particularly after hours. Decks could be created for this target audience in subjects such as pneumonia and fractures, respectively.

There are limitations to the extent of radiology that can be covered with our platform. While we believe our platform can eventually be expanded to include both basic imaging patterns targeted to beginning residents as well as more advanced categories, it would be extremely challenging in this format to master all relevant pediatric radiology knowledge. Moreover, our platform has a limited setup for textual information. Thus, while learners may be able to identify and characterize typical pediatric elbow fractures, this platform will need to be supplemented by material that explains the typical mechanism of injury, ages of patients and other more in-depth information. Such supplemental information can be found in review articles, textbooks, didactic lectures and common online resources such as StatDx or Radiopaedia, and our decks allow for linking to additional resources. Additionally, there is a substantial amount of non-image-based factual information that radiology trainees are required to learn. However, there are many flashcard programs that have the capability to incorporate this information (such as Anki).

Technically, we have made several major advancements over existing radiology quiz programs. No online quiz or radiology learning program currently available provides the ability to manipulate full DICOM data sets as unknown cases without special software. We were able to create new software as a proof of concept for integrating the PACS viewer from the Pacsbin platform directly into our spaced learning flashcards. This technology will allow us to further iterate on the concept of an embedded, interactive PACS and build more complete learning tools in the future. Further, we were able to create tools to leverage the PACS viewer to add quiz-specific functionality, such as region of interest-based questions. These let the quiz creator define image areas or volumes as correct for a given answer, allowing quiz takers to visually select an abnormality within the embedded PACS. These kinds of experiences are not generally available in existing quiz programs and allow more flexibility for educators and learners in designing a learning approach.

Practically, we have encountered multiple limitations to designing a website with a limited budget. Although the goals we proposed above have been included in the website, we continue to work through minor technical glitches commonplace to creating a website from scratch. For example, some of the outcomes data that we would like access to are currently only available to the developer via direct database queries, and additional work will need to be performed to make it easily accessible to the deck designer via an easy-to-use interface. We intend to use the available outcomes data to continually improve the decks as well as to refine the spacing algorithm. Monitoring utilization and performance of individual cards and decks will allow us to remove cards that cause confusion and improve decks that are underutilized. There are also improvements to be made to the flashcard authoring user interface. However, we were able to implement the desired core features, and this will be used as a foundation for further refinement before widespread implementation. Additionally, we plan to partner more closely with the Pacsbin platform in future iterations to provide a more streamlined flashcard authoring experience and develop additional tools for retrieving and managing outcomes data from quizzes. We are exploring options to obtain more funding or a collaborative partner to accelerate continued development. At this time, the site remains under development pending additional funding to incorporate the abovementioned modifications.

## Conclusion

Evidence from applied learning scientists has provided us with concrete strategies to improve long-term memory acquisition. Our platform primarily employs the strategies of self-testing and spaced learning to improve retention of key pediatric radiology knowledge by trainees, allowing them to create and share their own flashcard decks with PACS-like functionality. The tools and functional concepts we have created provide the foundation for a unique and powerful radiology learning platform.
